# Assessment of an evaluative tool for nursing college students: the tests for Objective Structured Clinical Examination (OSCE)

**DOI:** 10.1186/1472-6963-14-S2-P96

**Published:** 2014-07-07

**Authors:** Montserrat Sola-Pola, Anna M Pulpón-Segura

**Affiliations:** 1 School of Nursing, University of Barcelona, Barcelona 08907, Spain

## Background

The OSCE test is a tool used to assess the competence of Health Sciences’ students in a planned and structured way. It assesses their clinical skills based on simulations, specially designed according to the test objectives. More than 1,800 nursing students took an OSCE test which was implemented in 13 schools of nursing from to 2001 to 2011 in Catalunya.

## Objective

To assess the efficiency of an OSCE test from the students’ and lecturer’s point of view. The test has been done over ten years as part of the university nursing training.

## Materials and methods

A qualitative research method of the Grounded Theory was used. The tools chosen to collect information were a questionnaire with open questions, a focus group with students, and personal interviews with faculty. The method of constant comparisons and the software Atlas-ti were used to analyze the data.

## Results

Professors and students almost unanimously agree about the credibility of the staging and the contents of the situations posed in the test. It is positive that the simulations are carried out in a healthcare environment. However, some students manifest that it bothers them and they feel uncomfortable to act in front of professors who evaluate them. Students and professors agree on rating the content of the OSCE as appropriate in relation to the nurses’ role and representative of different areas of nursing practice. In general, the students believe that the difficulty of the test is reasonable. Coinciding with results from other studies [2], students considered that the OSCE test is stressful but a worthwhile experience.

## Conclusions

The OSCE test has been evaluated as an effective tool to assess skills in nursing students. Previous test information must be improved to decrease the stress generated by the students.
